# Awareness of Fixed Partial Dentures and Implant Rehabilitation of Missing Teeth Among a Subset of Saudi Population

**DOI:** 10.7759/cureus.33383

**Published:** 2023-01-05

**Authors:** Mahesh Suganna, Sara Tarek Ahmed, Hina Kausher, Abbasi Begum Meer Rownaq Ali, Yasmine Tarek Ahmed, Lulu Almuhaysh, Maryam Yousef, Reem Albgomi

**Affiliations:** 1 Prosthodontics, Riyadh Elm University, Riyadh, SAU; 2 Prosthodontics, College of Dentistry, Riyadh Elm University, Riyadh, SAU; 3 Prosthodontics/Dental Lab Technology, College of Applied Medical Sciences, Riyadh Elm University, Riyadh, SAU; 4 Prosthodontics, College of Applied Medical Sciences, Riyadh Elm University, Riyadh, SAU; 5 Restorative Dentistry, College of Dentistry, Riyadh Elm University, Riyadh, SAU; 6 Dental Lab Technology, College of Applied Medical Sciences, Riyadh Elm University, Riyadh, SAU

**Keywords:** questionnaire survey, prosthodontics, implant rehabilitation, fixed partial dentures, dental implants

## Abstract

Background

Maintaining one's sense of self requires having healthy teeth. A person's physical well-being is greatly impacted by their dental health. They are intimately related, and the socioeconomic situation of the individual largely determines how teeth are maintained. As a result, tooth loss causes injury to the stomatognathic system as well as the masticatory function. Morale is negatively impacted by psychological discomfort as well as the reduction in general quality of life brought on by tooth loss.

Objectives

The purpose of this study was to assess the awareness of patients about various dental prosthetic rehabilitative procedures in Saudi Arabia, their preference(s) regarding the choice of treatment, and the motivating factors that drive them to avail of dental prosthetic rehabilitative treatment.

Methods

After randomly selecting 600 individuals for the purpose of our investigation, a nine-variable questionnaire was framed by investigators to record the responses of those who consented to participate in our study.

Results

Only 68.3% of the respondents were found to be aware of the several prosthodontic replacement choices. As mentioned by the majority of the respondents, the cost element was the biggest drawback for replacement. The benefits of choosing fixed partial dentures (FPD) or dental implants were judged to be aesthetics (41.1%) and the feel of one's own teeth (40.1%).

Conclusion

Only 68.3% of respondents reported knowing about the several prosthodontic replacement choices. The cost aspect was cited by 348 respondents as the biggest drawback to replacement. The perceived benefits of choosing FPD or dental implants were deemed to be aesthetics (41.1%) and the feel of one's own teeth (40.1%). We believe that patients' health and quality of life can be improved by raising awareness about and changing patients' attitudes toward the most cutting-edge treatment options that are readily available. This can be done by educating people about the drawbacks of delaying the replacement of missing teeth and other treatment options.

## Introduction

A person's teeth are crucial to sustaining their sense of self. An individual's dental health status has a significant impact on their overall health [1]. They are intimately connected, and the individual's socioeconomic situation largely determines how teeth are maintained. An individual's quality of life is negatively impacted by psychological distress, social isolation, and physical damage brought on by tooth loss [2]. According to a study, there is an antagonistic association between social relationships, self-confidence, and missing or poorly fixed teeth [3]. In order to improve oral health, appearance, and self-confidence, tooth restoration is crucial. Dental caries, periodontal disease, dental trauma, or any other cause may result in tooth loss, but these factors will have an impact on daily activities like speaking and eating [4].

These days, losing one's teeth is viewed as a significant life event at both social and psychological levels [5]. Determining patient's thoughts toward the new dentures as well as whether he or she has had any negative effects from being edentulous for a long period will help the patient adopt the new dentures. In addition to enhancing patient comfort and masticatory performance, replacing missing teeth also helps to maintain the health and integrity of the dental arches, which in turn improves the patient's self-esteem [6]. Due to a variety of factors, including dental factors, patient factors, and prosthesis-specific factors, the care of partially edentulous patients with fixed partial dentures (FPD) poses ongoing challenges [7-9]. The prosthesis's components are stressed, and this tension can also cause problems in the supporting supports [10]. Abutment teeth are stressed during prosthesis use, insertion, and removal because they act as the prosthesis's supporting and holding components. If this force was greater than their natural capacity to withstand it, this could cause the supporting alveolar bone to resorb, the abutment to fracture, and ultimately the prosthesis to fail [11,12]. Similarly to this, when free-end saddle prostheses are used, they are stressed, which causes bone resorption, a loss of support, and a loss of stability, necessitating regular replacement. In the current situation, dentists have three options for replacing one or more lost teeth: dental implants, fixed partial dentures, or removable partial dentures.

The excellent predictability of oral implants has led to a change in the models for treating edentulous patients in recent years. In the past, dental implants were placed in patients who were completely denture-free in an effort to improve the stability of full denture prosthetics [13]. However, different dental implant loading methods have been presented, widening the range of implant rehabilitation programs for patients with partial dentures as implant treatment has become more predictable. Up to 90% of patients who receive implants today are partially dentate patients who need to be restored to health. Patients base their decision-making on socioeconomic, educational, and cultural perspectives [14]. Traditional fixed partial dentures and implants continue to be important tools in prosthodontics because of their significant durability and acceptable retention, even if implants need surgery and can be more expensive than fixed partial dentures [15]. In order to plan, implement, and evaluate practices toward fixed partial dentures and implant rehabilitation among the Saudi population, we wanted to provide baseline data and identify gaps through this survey. Our goals included determining how well the general public understood the different dental prosthetic rehabilitation techniques that are available, their preferences for the treatments they receive, and the factors that influence their decision to receive dental prosthetic rehabilitation.

## Materials and methods

Study design and domain

Ours was a cross-sectional, closed-ended, email-based questionnaire-based survey, with participation open to the entire Saudi Arabian population. A convenient random sampling method was employed, where the participants were randomly emailed the questionnaire, and all the study subjects’ voluntary participation and confidentiality were ensured.

Ethical consideration

The study was in compliance with the codes of research ethics from the Institutional Review Board (IRB) of Riyadh Elm University (REU), with IRB approval number FRP/2022/459/817/782 and registration number FRP/2022/459/817.

Sample size

The Raosoft® program (Raosoft Inc, Seattle, WA, USA) was used to determine the sample size. At the time this study was being conducted, the population of Saudi Arabia was estimated to be 36 million, comprising all five regions: the Eastern, Central, Northern, Eastern, Western, and Southern regions [16]. The required sample size was calculated at a 95 percent confidence level with an expected 50% response distribution and a margin of error of ±4% [17].

The null hypotheses were deemed valid if there was no significant variation observed in Saudi Arabian adults’ awareness of prosthodontic rehabilitation of missing teeth. There was no difference observed in opting for rehabilitation of missing teeth with FPD, implants, or removable partial dentures. There were no factors that affected the choice of FPD or implants for the rehabilitation of a missing tooth. The alternative hypotheses were deemed valid if there was variation observed in Saudi Arabian adults’ awareness of prosthodontic rehabilitation of missing teeth. There was a difference observed in the options observed in the study population for rehabilitation of missing teeth: FPD, implants, and removable partial dentures. There were factors that affected the choice of FPD and implants for the rehabilitation of missing teeth over a removable partial denture. After obtaining approval from the Institutional Review Board (IRB) of Riyadh Elm University (REU) during the month of February 2022, our study began. After observing that we had complied with the ethical requirements of the IRB at Riyadh Elm University, our investigation was approved with the IRB approval number.

Inclusion and exclusion criteria

Patients with both missing and normal teeth who were older than 18 years, without any systemic illness, and who consented to participate in this study were included. Patients who were ill, had other dental problems, were unwilling to participate in the study, or were under the age of 18 were excluded from the scope of our study. The questionnaire clearly mentioned our inclusion and exclusion criteria as mentioned above so as to not cause any confusion at the end of the respondent and ensure their convenience. The data obtained were subjected to statistical analysis using IBM SPSS Statistics for Windows, Version 23 (Released 2015; IBM Corp., Armonk, New York, United States). Data comparison was done by applying specific statistical tests to find out the statistical significance of the comparisons. To test for awareness between implants and FPD among the Saudi Arabian population, the Kolmogorov-Smirnov test was performed to determine the normality of the data. The test showed no significant differences, which confirmed that the obtained data were normally distributed. Variables were compared using numbers and percentages. The chi-square test was used to determine differences between groups based on Saudi Arabia's location. A P- value less than 0.05 was considered to be statistically significant. A nine-variable questionnaire was framed by the investigators. The validity of the questionnaire was tested on the following grounds: It was tested by expert validation assessed by Cohen’s Kappa. An expert panel of five members was tested. A value of 0.82 was found, suggesting acceptable inter-rater agreement for the items of the questionnaire by the experts. After checking for the reliability of the questionnaire, the same panel evaluated its adequacy and sufficiency in measuring the questionnaire. Every item was deemed necessary by all of the experts. The content validity ratio (CVR) was evaluated using Lawsche’s method, calculated using the formula,

CVR = ne - (N/2)

 N/2,

where ne is the number of expert panel members indicating "essential," and N is the total number of expert panel members. All nine items were scored on a range of 1 to 3 as "not necessary," "useful but not essential," and "essential." The final score of the CVR was 0.86. All the items were scored as "essential," and hence all questions or items were included in the final questionnaire.

The CVI was scored for each item. The item-content validity index (I-CVI) for all nine items was scored in the range of 0.95 - 1, thus suggesting 100 percent agreement. The scale level-content validity index (S-CVI) was based on the summary of all the I-CVI, which was scored at 0.88, thereby making it a relevant questionnaire.

## Results

We recorded 581 individuals who responded to our survey out of the 600 that we had expected as per our estimated sample size calculations. It was noted that only 68.3% of the respondents were aware of the various options for prosthodontic replacements. The greatest disadvantage felt for replacement was the cost factor reported by 348 of the respondents. Aesthetics (41.1%) and the feel of own teeth (40.1%) were thought to be the advantages of opting for FPD or dental implants. No significant difference was noted between the different locations of Saudi Arabia, which was categorized for the sake of analysis, since the respondents were clearly asked about their respective region by means of the questionnaire (Table 1).

**Table 1:** Variables assessing awareness for FPD or implants *: Significant; NS: not significant; df: degree of freedom; FPD: fixed partial denture

Variable	North N (%)	South N (%)	East N (%)	West N (%)	Central N (%)	Total N (%)	Chi square test	df	P value
Do you have any missing tooth in your mouth?									
Yes	15 (55.6)	45 (64.3)	150 (47.6)	22 (51.2)	55 (43.7)	287 (49.4)	8.734	4	0.066 (NS)
No	12 (44.4)	25 (35.7)	165 (52.4)	21 (48.8)	71 (56.3)	294 (50.6)			
Are you aware of various options for prosthodontic replacement of missing tooth?									
Yes	18 (72.0)	51 (76.1)	196 (66.9)	28 (66.7)	75 (67.0)	368 (68.3)	2.460	4	0.652 (NS)
No	7 (28.0)	16 (23.9)	97 (33.1)	14 (33.3)	37 (33.0)	171 (31.7)			
Do you know when a missing tooth should be replaced?									
Yes	5 (19.2)	16 (23.5)	62 (20.9)	6 (14.6)	22 (18.8)	111 (20.3)	3.914	8	0.865 (NS)
No	17 (65.4)	39 (57.4)	192 (64.9)	28 (68.3)	72 (61.5)	348 (63.5)			
If gaps are seen	4 (15.4)	13 (19.1)	42 (14.2)	7 (17.1)	23 (19.7)	89 (16.2)			
What are the disadvantages in using prosthodontic replacements?									
Cost	17 (70.8)	48 (76.2)	184 (65.9)	32 (80.0)	67 (63.8)	348 (68.1)	14.780	12	0.254 (NS)
Didn’t feel the need	4 (16.7)	6 (9.5)	35 (12.5)	2 (5.0)	9 (8.6)	56 (11.0)			
Lack of awareness	3 (12.5)	9 (14.3)	56 (20.1)	5 (12.5)	24 (22.9)	97 (19.0)			
No time	0 (0.0)	0 (0.0)	4 (1.4)	1 (2.5)	5 (1.0)	10 (2.0)			
Type of teeth you are keen on replacing when a particular tooth is damaged.									
Anterior teeth (front teeth)	8 (32.0)	18 (27.3)	80 (28.1)	7 (17.1)	27 (23.7)	140 (26.4)	6.730	8	0.566 (NS)
Posterior teeth (back teeth)	1 (4.0)	10 (15.2)	28 (9.8)	3 (7.3)	13 (11.4)	55 (10.4)			
Both	16 (64.0)	38 (57.6)	177 (62.1)	31 (62.1)	74 (64.9)	336 (63.3)			
Advantages of using FPD or dental implants									
Aesthetics (looking nice)	9 (40.9)	15 (24.2)	122 (46.6)	15 (40.5)	40 (37.7)	201 (41.1)	20.682	8	0.008*
Feels as your own teeth	4 (18.2)	33 (53.2)	100 (38.2)	16 (43.2)	43 (40.6)	196 (40.1)			
Less annoying	9 (40.9)	14 (22.6)	40 (15.3)	6 (16.2)	23 (21.7)	92 (18.8)			
How do you describe dental implants?									
Heard but cannot explain	10 (40.0)	19 (28.4)	90 (31.4)	14 (34.1)	33 (28.9)	166 (28.9)	11.057	12	0.524 (NS)
Metal piece	1 (4.00	3 (4.5)	9 (3.1)	5 (12.2)	5 (4.4)	23 (4.4)			
Screw	10 (40.0)	36 (53.7)	159 (55.4)	18 (43.9)	64 (56.1)	287 (53.7)			
Never heard	4 (16.0)	9 (13.4)	29 (10.1)	4 (9.8)	12 (10.5)	58 (10.9)			
How long does an Implant last?									
Between 5 and 10 years	4 (16.0)	8 (11.9)	35 (12.2)	4(9.8)	15 (13.2)	66 (12.3)	15.638	12	0.208 (NS)
More than 10 years	3 (12.0)	5 (7.5)	23(8.0)	2 (4.9)	16 (14.0)	49(9.2)			
Life time	3 (12.0)	27 (40.3)	81 (28.1)	11 (26.8)	37 (32.5)	159 (29.7)			
Not Sure	15 (5.7)	27 (40.3)	149 (51.7)	24 (58.5)	46 (40.4)	261 (48.8)			
Are you aware of complications present in prosthodontic replacement?									
Yes	10 (37.0)	34 (50.0)	98 (34.5)	20 (47.6)	39 (34.2)	201 (37.6)	11.494	8	0.175(NS)
No	5 (18.50	11 (16.2)	59 (20.8)	10 (23.8)	31 (27.2)	116 (21.7)			
May be	12 (44.4)	23 (33.8)	127 (44.7)	12 (28.6)	44 (38.6)	218 (40.7)			

## Discussion

In some countries, the media serves as the primary source of knowledge, whereas in others, the primary source of knowledge is the specialized practitioner. It can be theorized that community and ethical views and beliefs have an impact on people's decisions to receive prosthodontic treatment, particularly when it comes to aesthetics. Planning for the optimal FPD is necessary, and that process begins with a sufficient diagnostic impression and diagnostic casts [18]. The diagnostic cast is required to offer the dentist a thorough picture of the patient's condition, the prospective abutments' conditions and their inclination, the conditions of the opposing dentition, and the existence and specifics of the wear facets. This aids in the diagnosing process as well. Even in cases [19,20] where aesthetics is a top priority, the development of suitable augmentation procedures and the introduction of innovative implant surfaces have led to acceptable treatment outcomes [8]. In this regard, prosthetic and surgical methods have enhanced the aesthetic results for the teeth that will be replaced [21,22]. An outstanding aesthetic result is viewed from the patient's perspective as a suitable end to their dental issues. However, it is well recognized that biological issues can occur after dental implant installation, and infections can spread and necessitate a difficult, expensive peri-implant infection treatment. Once more, the patient is typically not aware of the biological dangers connected to implant implantation. In this setting, assessing treatment success in prosthodontics is increasingly dependent on patient perceptions and psychological factors. A growing number of recent studies on patient-reported outcome metrics reflect this [23,24].

Dental health has an effect on patients' overall health as well as their psychological well-being. Losing a tooth causes a variety of issues, including difficulty in chewing, aesthetic issues, speech issues, emotional stress, and social effects. People want to replace their missing teeth for these reasons. Implants and fixed prostheses are popular therapeutic choices. A couple of studies were observed to be based on somewhat similar methodological approaches to ours [25,26]. The current study, however, differs from others in terms of its scope, goals, sample, and location. Age, expense, time, and treatment anxiety are some of the variables that patients take into consideration when deciding whether to have a lost tooth replaced [15,27,28]. In agreement with a study by Osterberg et al., who found that aesthetic rather than functional grounds was the requirement for tooth replacement, subjects in the current study indicated function as the primary reason for the replacement of a missing tooth [29]. A significant factor that was considered in this study was the location of the lost tooth, which 64% of our respondents reported as a significant factor. This was in line with what Hussain et al. discovered that the location of the restoring tooth has a significant impact on the decision to undergo treatment [30]. For example, all patients prefer to treat their anterior teeth for aesthetic reasons rather than restore their posterior teeth for functional reasons. In our study, 68.3% of the respondents were aware of the various options for prosthodontic replacements, but 63.5% of the individuals were not sure when to replace the missing tooth, which is in contrast to the study by Akeel et al., which found that 76.2% of his study sample in Saudi Arabia had positive attitudes and knowledge towards replacing a missing tooth [31]. Hussain et al. [30] reported that younger patients preferred implant-supported prostheses to other prostheses, comparable to the study by Abdurahiman et al. and Schützhold et al. [32,33], where age played a significant influence, a fact that we overlooked in our study for the sake of respondent convenience. In contrast to Hussain et al., we did not account for gender-specific motivations related to the replacement of lost teeth [30]. The preferred course of treatment, according to a set of studies, is surgery, a figure that somewhat correlates with our findings, where 41% of the individuals accepted knowing about implants for aesthetic-related improvements, and 40% of our respondents had reportedly heard about implants but did not know exactly what an implant was [34,35]. This might be a result of population shifts in socioeconomic status and literacy levels. The findings of our study show that 53.7% of individuals had moderate awareness levels concerning dental implants, a statistic vaguely similar to the findings observed by Pommer et al. [36] and Al-Johany et al. [37].

In terms of limitations, not taking into account variables such as gender-related motivations pertaining to replacement of tooth loss, socioeconomic data, and educational information about the respondents could be one major flaw in our study, but due to respondent-related convenience (so that answering the questionnaire did not take much time), we overlooked such variables. Also, with the population size of the area that we targeted for our study, the number of actual respondents that relayed our questionnaire, i.e., our sample size, back to us was somewhat small in comparison to other questionnaire- or survey-based studies performed. However, because ours was one of the few studies in this part of the world that attempted to establish some kind of correlation between awareness of fixed partial dentures and implant rehabilitation of missing teeth, we believe that more studies could be conducted using similar methodological approaches that we used to validate the results that we obtained. 

## Conclusions

Our analysis clearly shows that most respondents were aware of the numerous prosthodontic replacement choices. The expense was considered the biggest drawback for replacement, but the aesthetics and natural feel of one's own teeth were considered positives for choosing FPD or dental implants. The greatest disadvantage assessed for the replacement of teeth in our investigation was the cost factor, with aesthetics and the sentiment associated with individuals having their "own" teeth contributing significantly to the advantages of opting for FPD or dental implants in this study. We believe that through studies like these, people can be made aware of the disadvantages caused by the immediate non-replacement of missing teeth and also about other treatment options so that patients’ health and quality of life can thereby be improved by increasing their awareness and improving their attitude toward the most advanced treatment options that are readily available.
